# Editorial: The Translational and Therapeutic Potential of the Tumor Microenvironment in Oral Cancer

**DOI:** 10.3389/froh.2021.763731

**Published:** 2021-09-27

**Authors:** Keith D. Hunter, Daniel W. Lambert, Ricardo D. Coletta

**Affiliations:** ^1^The School of Clinical Dentistry, The University of Sheffield, Sheffield, United Kingdom; ^2^Department of Oral Diagnosis, School of Dentistry, University of Campinas, São Paulo, Brazil; ^3^Graduate Program in Oral Biology, School of Dentistry, University of Campinas, São Paulo, Brazil

**Keywords:** tumor microenvironment, oral cancer, inflammation, fibroblasts, therapeutic potential

It is now recognized that the tumor microenvironment (TME) can influence all the essential hallmarks of cancer, contributing to growth, progression and treatment response of tumors. The TME is composed of many cellular components including a vast repertoire of immune/inflammatory cells, blood and lymphatic vessels, peripheral nervous structures, fibroblasts and an array of non-cellular components such as extracellular matrix proteins, cytokines and growth factors. As the TME is a crucial part of the tumor, its components have become one of the key targets for tumor treatment. Although many strategies to target the TME have been developed, the results so far are disappointing, with limited impact observed on tumor burden or overall survival. This special collection encompassed 7 reviews and 2 original articles dedicated to the translational and therapeutic potential of the TME in oral cancer, highlighting opportunities and the need to concentrate efforts to overcome the barriers to develop new and efficient TME-targeted therapeutic strategies.

Several of the studies discuss emerging findings related to the tumor-infiltrating immune/inflammatory cells. It is becoming clear that a cause-effect relationship exists between inflammation and cancer, and Elebyary et al. performed an extensive review in the putative connection between oral carcinogenesis and the chronic inflammation associated with periodontitis. The authors provided evidence that the inflammatory milieu in periodontitis is ideal for cancer cell seeding, migration, proliferation and immune escape. Moreover, the authors showed evidence that dental biofilm (bacterial)-derived substances may contribute to the induction of permanent genomic alterations. Niklander extensively reviewed the literature to characterize the major TME-associated inflammatory cells and mediators that may contribute to proliferation and spread of oral cancer cells. This study also highlighted the rationale for detection of salivary inflammatory factors as biomarkers for the diagnosis of oral squamous cell carcinoma (OSCC).

Evasion of immune surveillance and induction of an immunosuppressive TME are common features of the head and neck squamous cell carcinomas (HNSCC), and one of the main immune escape mechanisms includes the overexpression of the programmed death ligand-1 (PD-L1) in the surface of the tumor cells. The article by Wondergem et al. reviewed the currently approved immune checkpoint inhibitors (drugs that block immune checkpoint proteins such as PD-L1) in HNSCC, including nivolumab and pembrolizumab (anti-PD-1 antibodies), and highlighted the potential benefits of modulating STAT3 and in PI3K/AKT/mTOR and Wnt pathways to boost the response to these inhibitors and prevent drug resistance. In this context, Dobriyan et al. reported the differences in the immune/inflammatory cells and cancer-associated fibroblasts (CAFs) between patients with OSCC treated with neoadjuvant pembrolizumab that displayed complete or incomplete remission. In the patients with complete remission, the tumor was replaced by a granulomatous type of inflammation, enriched with T lymphocytes, with approximately equal amounts of CD4^+^ and CD8^+^ cells, numerous CD68^+^ and CD163^+^ macrophages and absence of CAFs. The tumors with incomplete remission showed a moderate inflammatory response, with a variable CD4^+^/CD8^+^ ratio, and the presence of CAFs. These encouraging results warrant further investigation in large cohorts.

Hypoxia is an important feature of the TME, and the review by Chaudhary et al. explored its effects on the crosstalk between OSCC cells and immune cells. The authors reported that in the hypoxic microenvironment, OSCC cells and other TME components secrete immunosuppressive onco-metabolites that regulate immune escape, via disturbing redox balance, mitochondrial function and ATP production through aerobic glycolysis.

The connection between inflammation and oral carcinogenesis was also reviewed in Niklander et al. by exploring the roles of interleukin 1 (IL-1) signaling, which has been shown to be activated in several types of tumors, in HNSCC. After reviewing important roles of the main IL-1 family members in the growth, differentiation and aging of normal oral keratinocytes, the authors showed that the dysregulated expression of IL-1 and IL-1R are associated with the acquisition of malignant phenotypes and reduced survival in HNSCC. The authors also provided evidence of the potential of IL-1 family members as diagnostic biomarkers and as attractive therapeutic targets for HNSCC. The participation of the IL-1/IL-1R axis in oropharyngeal squamous cell carcinomas (OPSCC) was investigated in Al-Sahaf et al.. Initially the authors demonstrated that HPV-negative OPSCC contained significantly more neutrophils than HPV-positive tumors. Applying a 3D cell culture model with HPV-negative or HPV-positive OPSCC cells in the presence of anakinra, an IL-1R inhibitor, the authors showed that both chemokine secretion and neutrophil recruitment are dependent on IL-1β/IL-1R paracrine signaling. These features make IL-1/IL-1R axis promising biomarkers and therapeutic targets for HNSCC.

The role of CAFs in OSCC is extensively reviewed in Bienkowska et al.. Besides summarizing our current understanding of CAF subtypes and function in controlling tumor proliferation, invasion, metabolic switch, angiogenesis, immune surveillance and therapy resistance, which all contribute to poor patient survival, the article discusses CAF-targeting therapies. The potential strategies discussed in the article include inhibiting CAF activation or function (e.g., inhibiting TGF-β or CXCL12/CXCR4 signaling pathways), “normalizing” CAF (e.g., NOX4 inhibitors) or killing CAF (e.g., FAP-based depletion). Although these strategies are promising, caution is required because those molecules are widely expressed in different conditions and tissues and their targeting may result in many side effects.

The participation of the sympathetic nervous system in the development and progression of HNSCC was explored in Vincent-Chong and Seshadri, by investigating the neurovascular interactions mediated via the adrenergic signaling. This study also brought a basis to support the therapeutic potential for HNSCC of directly targeting adrenergic signaling in tumor cells or indirectly targeting neurovascular interactions.

This Research Topic is a useful reference of the current understanding about the vital role of TME in oral cancer development and progression, highlighting its potential to development of new strategies for oral cancer treatment.

## Author Contributions

All authors listed have made a substantial, direct and intellectual contribution to the work, and approved it for publication.

## Conflict of Interest

The authors declare that the research was conducted in the absence of any commercial or financial relationships that could be construed as a potential conflict of interest.

## Publisher's Note

All claims expressed in this article are solely those of the authors and do not necessarily represent those of their affiliated organizations, or those of the publisher, the editors and the reviewers. Any product that may be evaluated in this article, or claim that may be made by its manufacturer, is not guaranteed or endorsed by the publisher.

